# Single breath count test and its applications in clinical practice: a systematic review

**DOI:** 10.1097/MS9.0000000000001853

**Published:** 2024-02-28

**Authors:** Samikchhya Keshary Bhandari, Anil Bist, Anup Ghimire

**Affiliations:** Tribhuvan University Institute of Medicine, Maharajgunj Medical Campus, Internal Medicine, Maharajgunj, Nepal

**Keywords:** myasthenia gravis, single breath count test, spirometry, systematic review

## Abstract

**Background:**

Single breath count test (SBCT) may be a reproducible, rapid, easy to perform and easy to interpret substitute to spirometry especially in low resource settings for certain conditions. Its interest has been rekindled with the recent COVID-19 pandemic and it can be done as a part of tele-medicine as well.

**Objectives:**

The objective of this review was to summarize the evidence of SBCT in clinical practice.

**Methods:**

The authors searched EMBASE, PubMed and Google Scholar for all the relevant articles as per exclusion and inclusion criteria. Two authors independently screened all the studies. Newcastle Ottawa Scale was used to assess the quality of the studies. The systematic review was carried following the PRISMA guidelines.

**Results:**

After the rigorous process of screening, a total of 13 articles qualified for the systematic review. SBCT greater than 25 had sensitivity of greater than 80% in diagnosing myasthenia gravis exacerbation and SBCT less than or equal to 5 predicted the need for mechanical ventilation in Guillain–Barre syndrome (GBS) patients with 95.2% specificity. Also, Single breath count correlated significantly with forced expiratory volume in 1 sec (FEV1) and forced vital capacity (FVC) in children with pulmonary pathology and in patients with COVID-19 it was used to rule out the need for noninvasive respiratory support.

**Conclusion:**

SBCT will undoubtedly be an asset in low resource settings and in tele-medicine to assess the prognosis and guide management of different respiratory and neuromuscular diseases.

## Introduction

Highlights

- Single Breath count test is a easy to Perform and cost-effectiveness measure.
- Single breath count has very high sensitivity in evaluating Pulmonary function.
- Single breath count test can be carried out in resource limited setting as part of tele-medicine to assess severity of multiple pathologies.

Continuous monitoring of the respiratory function of patients with neuromuscular disease is important. Although easily available, the measurement of oxyhemoglobin saturation using pulse oximeters is not an ideal method for monitoring patients with increased risk of neuromuscular disease related respiratory compromise. This is because the oxyhemoglobin dissociation curve is sigmoidal in shape due to a phenomenon called positive cooperativity. Positive cooperativity is a phenomenon characterized by the increase in affinity of haemoglobin for oxygen with the increase in the number of oxygen molecules bound to it^1^. This sigmoidal shape causes the haemoglobin oxygen saturation to be maintained even when partial pressure of oxygen is decreasing in the blood. This is the reason why the measurement of oxygen saturation in blood may be a lagging indicator of true oxygenation. Due to this reason, using oxyhemoglobin saturation levels of haemoglobin may cause a delayed, and sometimes life-threatening, recognition of hypoxia.

Spirometry is a gold standard test to measure the mechanical pulmonary function which is necessary for diagnosing and management of several diseases. However, spirometry requires well trained and experienced testing personnel^2^ along with accessibility to a standardized device^3^ which is not widely available in all low resource settings.

The alternative, for oxyhemoglobin saturation measurement as a predictor of hypoxia in neuromuscular diseases, is the use of spirometry. As spirometry indices measure severity of the root cause of hypoxia that is respiratory muscle inefficiency rather than the downstream effect of that inefficiency that is the hypoxia, can be considered as a leading indicator of respiratory compromise and need of mechanical ventilation^4^.

It has been shown that the probability of obtaining a good quality spirometry test ranges from 35 to 60% despite using standardized spirometers with frequent quality checks^5,6^ In addition, interpretation of wide range of spirometry indices is challenging and requires a specialized education in the field^7^.

Unfortunately, performing spirometry is time intensive, cumbersome and it may not be available widely especially in low resource settings. Low quality spirometry assessment is common secondary to various issues with the equipment, patient, technologist or interpreter^8^.

Single breath count test (SBCT) may be a reproducible, rapid, easy to perform and easy to interpret substitute to spirometry especially in low resource settings for certain conditions. Its interest has been rekindled with the recent COVID-19 pandemic and it can be done as a part of tele-medicine as well. SBCT may be a useful alternative to pulmonary function test (PFT) as it is easy to perform, reproducible, fast, without economic costs and without the risk of spread of infections via cross contamination of equipment.

In this systematic review, we aim to summarize the evidence regarding the use of SBCT in management of various pathologies in clinical practice.

## Materials and methods

### Protocol and registration

This review was conducted in accordance with the Preferred Reporting Items for Systematic Reviews and Meta-Analyses (PRISMA)guidelines, Supplemental Digital Content 1, http://links.lww.com/MS9/A384 ^9^ and reported in line with AMSTAR, Supplemental Digital Content 2, http://links.lww.com/MS9/A385 ^28^. The protocol has been registered in PROSPERO international prospective register of systematic reviews.

### Inclusion criteria

1. Eligible study types: All types of studies including but not limited to case reports, case series, case-control studies, cross-sectional studies, cohort, randomized controlled trials or viewpoints.
2. Eligible study participants: Patients with all pathologies and all age groups will be included.
3. Eligible study interventions: Use of SBCT for management of any condition.
4. Eligible study comparison: Not applicable for our study.

### Exclusion criteria

Articles reported in languages other than English. All the articles not reporting the variables and outcome of interest.

### Information sources and search strategy

Primary literature search was done through three electronic databases; MEDLINE (accessed through PubMed), EMBASE and Google Scholar from inception of the database to 12 May 2022 using the predefined keywords. Terms like “single breath count”, “single breath count test”, “Sbct”, “single breath counting” were used in all the databases to search for possible records. Secondary literature search was done by checking the reference of included studies.

### Study selection and data extraction

All the results were then imported in Endnote X7 (Thomson Reuters). The titles and abstracts of the references retrieved during the searches were screened and duplicates were removed by two authors and potentially relevant full-texts were then screened in pairs by authors. Data were extracted independently by two authors from the studies selected for inclusion onto a standardized Excel sheet. Any discrepancies were resolved through discussion with the third author. The search Strategy can be found in supplementary files section S1, Supplemental Digital Content 3, http://links.lww.com/MS9/A386.

Data were manually extracted from eligible studies in a standardized data extraction Excel Spreadsheet (Microsoft Corp). The following variables were extracted: author, type of study, year of publication, age of patients, sex, pathology, methodology of SBCT, and results of the respective studies.

### Quality assessment

Modified version of the Newcastle Ottawa scale was used for quality assessment of the final studies^10^ Ten studies scored 5 or more whereas 2 studies scored 4 in the Newcastle Ottawa scoring system. The detailed Newcastle Ottawa Scoring of included studies for quality assessment can be found in the supplementary files section S2, Supplemental Digital Content 3, http://links.lww.com/MS9/A386.

## Results

### Search results

**Figure 1:**
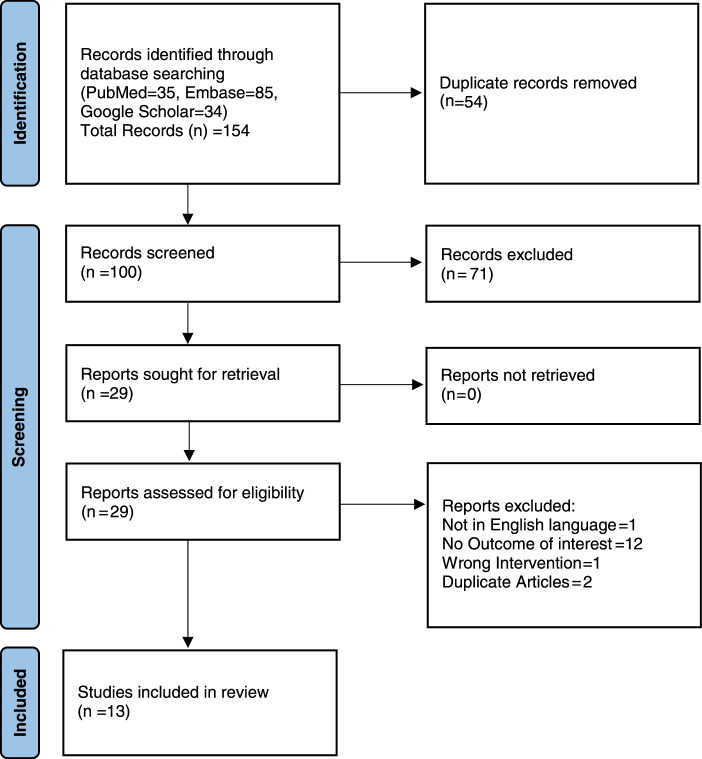
Preferred Reporting Items for Systematic Reviews and Meta-Analyses (PRISMA) flowchart showing the search and study selection.

The preliminary literature search yielded a total of 154 articles (85 articles from EMBASE, 35 articles from PubMed and 34 articles from Google Scholar). After removal of 54 duplicate articles, remaining 100 articles were screened for title and abstract, out of which 71 articles were excluded and 29 articles were selected for full text review. Out of 29 articles 16 articles didn’t fulfil the inclusion criteria and were excluded and remaining 13 articles which fulfilled the inclusion criteria were included in the review. The PRISMA flowchart depicting the detailed process of study selection is displayed in Figure 1.

### Study characteristics

A total of 13 studies (*n*=13) were included in the review out of which 10 studies were prospective studies^11–20^, 1 was retrospective^21^, 1 viewpoint^22^ and 1 was cohort^23^ altogether consisting of 1300 participants (sample size ranging from 22 to 516). Two studies^12,20^ recruited healthy patients, and the remaining 10 studies recruited patients with one or more illnesses. The detailed characteristics of each study are summarized in Table 1.

**Table 1:** Baseline characteristics of included studies.

Author	Country	Type	Sample size	Mean age (SD)	Sex	Pathology
Elsheikh *et al*., 2016^11^	NA	Prospective cross-sectional single blind study	31	57 (19)	16M/15F	Myasthenia gravis
Kukulka *et al*., 2020^21^	USA	Retrospective single centre review	25	42.9 (18.4)	10M/15F	Myasthenia gravis
Kumari *et al*., 2017^12^	India	Observational cross-sectional	183	Median age (IQR) 20 (19–21)	82M/100F	None (healthy adults)
Kalita *et al*., 2020^13^	India	Prospective	94	Median age 30 (range 6–70)	64M/30F	GBS
Bartfield *et al*., 1994^14^	USA	Prospective	22	Mean age (SD) 51 (18)	12M/10F	Asthma=4, Bronchiectasis=2, cough=3, dyspnoea=3, Interstitial lung disease=5, preoperative screening=5
Ali *et al*., 2011^15^	USA	Prospective observational study	67	Median age 12	43M/24F	Asthma=44, cystic fibrosis=9, chronic diseases=14
Longhitano *et al*., 2021^16^	Italy	Prospective observational study	120	Median age (SD) 66.9 (12.5)	72M/48F	COVID-19 pneumonia
Quinn *et al*., 2021^23^	USA	Cohort	30	Median age (IQR) 58 (49.25–73.25)	21M/9F	ALS
Kannan Kanikannan *et al*., 2014a^17^	India	Prospective Study	68	NA	47M/21F	GBS
Escóssio *et al*., 2019^18^	Brazil	Cross-sectional study	516	Median age (SD) 37(31.1)	270M/246F	Variety of diseases
Ushkow *et al*., 1998^19^	USA	Prospective study	44	Mean age (SD) 34(11.8)	15M/28F	Bronchospasctic
Rega *et al*., 2010^22^	USA	Viewpoint	NA	NA	NA	Botulism
Bhandare *et al*., 2021^20^	India	Cross-sectional	100	Mean age (SD) 31.54(10.52)	29M/71F	Healthy volunteers

ALS, Amyotrophic Lateral Sclerosis; F, female; GBS, Guillain–Barre syndrome; M, male; NA, not available.

### SBCT in healthy population

Only two studies recruited a healthy population in order to evaluate the SBCT. Kumari *et al*.^12^ conducted a study in 183 healthy volunteers with age range of 18–26 in order to determine the interquartile range of SBCT which ranged from 31 to 49. Bhandare *et al*.^20^. in a study among 100 healthy participants with a mean age of 31.5 years found that SBC had a significant correlation of 0.7 with peak expiratory flow rate (PEFR).

### SBCT in pulmonary pathology

In a study by Bartfield and colleagues comprising of 22 patients, with multiple pathologies, the value of SBCT in 6 patients with Obstructive lung disease had good correlation with forced expiratory volume in 1 sec (FEV1) (correlation coefficient=0.68) and 5 patients with interstitial lung disease (ILD) were found to have good correlation between SBCT and PEFR (correlation coefficient =0.63).

Similarly, Ushkow *et al*.^19^ found that 44 adult patients with a mean age of 34.3 with bronchospastic disease had correlation coefficient of 0.18 between PEFR and SBC. This is low compared to study by Bartfield *et al*.^14^. However, quality of studies by Bartfield and colleagues and Ushkow and colleagues were unsatisfactory with modified NOS of less than 5. Also the implication of SBCT over multiple age groups was demonstrated by the findings in study by Ali *et al*.^15^ which reports the significant correlation of SBCT with FEV1 (correlation coefficient=0.66) and forced vital capacity (FVC) (correlation coefficient=0.71).

### SBCT in myasthenia gravis

There have been multiple studies on the efficacy of SBCT in monitoring myasthenia gravis patients. Elseikh *et al*.^11^ found that SBCT showed significant correlation with FVC, Net inspiratory force (NIF) and neck flexor strength in 31 patients with acetylcholine receptor positive Myasthenia Gravis. Kukulka *et al*.^21^ found that SBCT was highly sensitive in determining the presence of MG exacerbation through telephonic assessment of SBCT with a cut-off value of 25 having a sensitivity of 80% and specificity of 60% in diagnosing MG exacerbation.

### SBCT in Guillain–Barre syndrome (GBS)

SBCT has been used in monitoring patients with GBS as well. In a study including 94 patients, Kalita *et al*.^13^ discovered that SBCT value of 5 could predict, with a sensitivity of 90.6% and specificity of 95.2%, the necessity for mechanical breathing. Furthermore, relative change in SBC at 24 h of less than 20% had a negative predictive value of 80.8% in ruling out the need for mechanical ventilation. Kanikannan *et al*.^17^ found that among 68 patients with GBS, SBC had significant correlation with FVC, FEV1 and phrenic nerve distal motor latency (DML) with correlation coefficient of 0.75, 0.74 and 0.453, respectively. In addition, SBC score of more than 19 had sensitivity of 95% in ruling out the need for mechanical ventilation.

### SBCT in ALS

SBCT has been used in monitoring patients with Amyotrophic Lateral Sclerosis as well. Quinn *et al*.^23^ found that in 30 patients, an externally paced SBCT of 11 had a sensitivity of 100% and specificity of 56% for predicting FVC less than 50%.

### SBCT in COVID-19

The utility of SBCT in triaging and monitoring of patients with COVID-19 seems to be promising. Longhitano *et al*.^16^ conducted a study on 135 patients and found that a SBCT cut-off value of 32 had a sensitivity of 92.3% and specificity of 57.1% in determining which patients require noninvasive respiratory support (NIRS). SBCT was found to be more sensitive than PaO2/Fio2 and respiratory rate (RR) in this regard. However, the specificity was lower compared to other tests. This implies that SBCT can be used for ruling out patients who do not require NIRS which can be extremely beneficial during triage. Similarly, Bhandare *et al*.^20^. found that SBCT correlated well with the improvement of patients in post-COVID period.

### SBCT in botulism and snake bite

Rega *et al*.^22^ have suggested that SBCT may be useful in triaging mass casualty of patients with botulism. In addition, there may be a role of SBCT in monitoring patients with neurotoxic snake bites as the pathophysiology of neurotoxic snake bite is similar to that of myasthenia gravis^24^.

### Characteristics of SBCT

All the studies^11–16,21,23^ used frequency of 2 Hz normal voice for SBCT except for^17,18,22^ which were unspecified. Two attempts were made in^11,21^ studies, and three attempts were made in^12–15,17,19,20^ and accordingly the final result of SBCT was calculated by average or best of the attempts in studies. Upright sitting position was also used in majority of the studies^12,13,16,18,21^ The intensity of vocalization was normal voice in all the studies except the Rega *et al*.^22^. which used the “count out loud”. The characteristics of SBCT in various studies is given below in Table 2.

**Table 2:** Characteristics of single breath count test.

Study	Frequency	Intensity of vocalization	Posture	No. attempts	Recording of result
Elsheikh *et al*., 2016^11^	2 Hz	Normal voice	Not specified	2	The better of two attempts was recorded
Kukulka *et al*., 2020^21^	2 Hz	Normal voice	Sitting upright	2	The better of two attempts was recorded
Kumari *et al*., 2017^12^	2 Hz	Normal voice	Sitting upright	3	The better of three attempts was recorded
Kalita *et al*., 2020^13^	2 Hz	Normal voice	Sitting upright or propped up at 70 degrees	3	The average of 3 readings was recorded
Bartfield *et al*., 1994^14^	2 Hz	Normal voice	Not specified	Maximum of 6	Repeated until 3 scores were within 10% of each other
Ali *et al*., 2011^15^	2 Hz	Normal voice	Not specified	3	The average of 3 attempts were recorded
Longhitano *et al*., 2021^16^	2 Hz	Normal voice	Sitting position	2	Not specified
Kannan Kanikannan *et al*., 2014a^17^	Not specified	Normal voice	Not specified	3	Not specified
Escóssio *et al*., 2019^18^	Not specified	Normal voice	Sitting position	3	The better of 3 attempts was recorded
Ushkow *et al*., 1998^19^	2 Hz	Normal voice	Not specified	Maximum of 3	Repeated until 2 measurements agreed to within 15%
Rega *et al*., 2010^22^	Not specified	“Count out loud”	Not specified	Not specified	Not specified
Bhandare *et al*., 2021^20^	2 Hz	Normal voice	Not specified	3	The better of 3 attempts was recorded
Quinn *et al*., 2021^23^	Patient paced	Normal speaking voice with numbers audibly distinct	Not specified	Not specified	Not specified
	Externally paced (2 Hz)	Normal speaking voice with numbers audibly distinct	Not specified	Not specified	Not specified

### Cut-off level for SBCT in multiple pathologies

Cut-off value of SBCT for various pathology was defined only in 6 articles^13,16–18,21,23^ for different pathologies. The cut-off value varied widely ranging from 5 to 32 in various pathologies across studies. Myasthenia Gravis exacerbation was correlated to the value of a SBCT below 25 with sensitivity of 80%^21^. Similarly SBCT value greater than 19 ruled out the need for the mechanical ventilation in the patients with Guillain–Barre Syndrome with sensitivity of 95% whereas the value of SBCT less than or equal to 5 strongly predicted the need for mechanical ventilation with 95.2% specificity. Similarly in Motor neuron disease Amyotrophic Lateral Sclerosis SBC value below 11 can be used to predict the declining vital capacity less than 50 with 100% sensitivity so that timely ventilatory support can be given^23^. SBCT also predicts the need for noninvasive ventilation support in patients with COVID-19 with 92.3% sensitivity due to which it can be used as alternative to the standard of spirometry in determining the respiratory variables. The cut-off value in different studies is given below in Table 3.

**Table 3:** Cut-off levels of single breath count test below which it signifies abnormal.

Study	Patient population	SBCT cut-off value	Implication
Kukulka *et al*., 2020^21^	Myasthenia gravis	25	SBCT < 25 had sensitivity of 80% in diagnosing myasthenia exacerbation
Kannan Kanikannan *et al*., 2014a^17^	GBS	19	SBCT>19 had sensitivity of 95% in ruling out the need for mechanical ventilation
Kalita *et al*., 2020^13^	GBS	5	SBCT<or=5 predicted need for mechanical ventilation with specificity of 95.2%
Quinn *et al*., 2021^23^	ALS	11	SBCT cut-off of 11 had sensitivity of 100 percent in predicting FVC<50%
Longhitano *et al*., 2021^16^	COVID-19	32	SBCT cut-off of 32 had sensitivity of 92.3% in determining patients requiring NIRS
Escóssio *et al*., 2019^18^	Variety of diseases	21	SBCT of 21 or less had a sensitivity and specificity of 94.4% and 76.62% for VC <20 ml/kg

ALS, Amyotrophic Lateral Sclerosis; GBS, Guillain–Barre syndrome; FVC, forced vital capacity; NIRS, noninvasive respiratory support; SBCT, single breath count test.

## Discussion

The SBCT is an easy-to-use bedside tool with potential utility in a variety of diseases, including pulmonary, neuromuscular, infectious, and poisonous illnesses. The aim of our systematic review was to summarize the existing literature and generate information of the wide utility of use of SBCT in clinical practice. Our findings imply that SBCT could be used to detect and monitor a variety of medical disorders, including but not limited to exacerbation of Myasthenia Gravis, respiratory pathologies such as asthma, cystic fibrosis, COVID-19, and snake bites.

Our review identified significant correlations between SBCT and multiple variables of pulmonary function, such as FVC FEV1, and PEFR in patients with various pathologies. Ushkow *et al*.^19^. reported the improvement in the SBCT values coupled with the improvement in PEFR (mean r=0.87) during the management of an exacerbation of reactive airway disease. SBC was also moderately correlated (r=0.66) to FEV1 in a by Similarly study by Dishinca *et al*.^27^. and Elsheikh *et al*.^11^. supported SBCT as a valid bedside test for MG exacerbation predictor as it strongly correlated with the values of spirometry. These results are corroborated by research by Octaviana *et al*.^25^ and.Aguirre *et al*.^26^. In a study by Kumar *et al*.^29^ for patients not receiving ventilator support, the median SBC upon admission was 24, whereas it was 10.5 for those receiving it indicating the possibility of use of SBCT as bedside measure of respiratory function to predict need of ventilator support when spirometers are not easily available.

SBCT also showed potential utility in forecasting the need for mechanical ventilation in the setting of COVID-19.The study by Longhitano *et al*.^16^ developed a cut-off value for SBCT that demonstrated a great sensitivity of 92.3% in identifying patients who needed NIRS. This information suggests that SBCT can be used as a simple and noninvasive quick technique to assess the respiratory state of COVID-19 patients, particularly during the pandemic when healthcare resources are under demand. In addition, there may be a role of SBCT in monitoring the effectiveness of “Neostigmine Test” in patients with neurotoxic snake bites as the pathophysiology of neurotoxic snake bite is similar to that of myasthenia gravis^24^.

SBCT exhibited variability in cut-off values between studies conducted by Kalita *et al*.^13^ and kanikannan *et al*.^17^. This variation may be attributed to differences in the methodology used for conducting the test, also the frequency used by Kanikannan and colleagues is unspecified which can have caused the interstudy variation in cut-off values. The variations in cut-off values caused by the various techniques utilized while performing a SBCT may make planning for intervention difficult, especially in settings where cross-validation methods are not readily available, thereby compromising its widespread adaptability. Likewise, validation in multiple languages is crucial due to phonetic differences in numerals and varying syllable counts across languages. Therefore, standardization of SBCT is crucial to ensure consistency and reliability of results. Similar to what the American Thoracic Society and the European Respiratory Society recommend for spirometry^3^, we suggest uniformity in the use of techniques for doing SBCTs. To reduce variation in test results, this standardization should cover a variety of test elements, such as patient position, vocal intensity, counting speed, and baseline respiratory function. Similar recommendations have been proposed^27^ for Myasthenia Gravis but the improvement in the sensitivity and specificity of the test while making it uniform haven’t been demonstrated in any studies which can be an opportunity to explore in further studies. The lack of an established reference range for healthy individuals is a significant hurdle in assessing the effectiveness of SBCT across various medical conditions. Studies have yet to explore the utilization of SBCT in cases approaching respiratory failure or its correlation with critical thresholds guiding ICU admission, intubation, and ventilation in such patient cohorts. A study by Kumari *et al*.^12^ attempted to establish a reference range in 183 healthy adults aged 18–26, resulting in values ranging from 31 to 49. However, this study’s limited sample size and narrow age range suggest the need for further research to determine a comprehensive reference range for SBCT in healthy populations. To optimize the utility of SBCT, further validation across diverse demographics, standardized protocols, comparative analyses against traditional tests, acknowledgement and mitigation of biases, establishment of robust reference ranges, exploration of clinical integration, and targeted future research directions are imperative.

Our study has several limitations that need to be addressed, including the inability to include articles published in languages other than English, the inclusion of studies including those with suboptimal quality, interstudy heterogeneity in the population, and variability in the method used to conduct SBCTs which may have affected study.

## Conclusion

The SBCT demonstrates significant potential as a versatile clinical tool for diagnosing and monitoring a wide array of medical conditions. Its simplicity, noninvasiveness, and cost-effectiveness make it a promising alternative to spirometry to evaluate pulmonary function in pulmonary pathologies, neuromuscular diseases and life-threatening conditions like snake bite, especially in underserved rural areas where access to spirometry expertise may be limited. The use of SBCT could lead to substantial cost savings and a more efficient utilization of healthcare resources, as it requires no specialized equipment beyond readily available timers or metronomes on common smartphones.

To ensure the accuracy and reproducibility of SBCT-based treatments across various clinical scenarios, further research is imperative. Precise reference ranges for SBCT values in healthy individuals need to be established. As research and development efforts progress, SBCT may evolve into an indispensable tool in the clinician’s arsenal for the early detection and management of a wide range of medical conditions.

## Ethical approval

The ethical approval was not needed because this study doesn’t directly deals with individual patients. This is a secondary analysis of published studies. It is a Systematic Review.

## Consent

Not applicable to our study because our study is analysis of secondary published studies.

## Source of funding

This research did not receive any specific grant from funding agencies in the public, commercial, or not-for-profit sectors.

## Author contribution

A.B.: literature search, draft writing, revision of manuscript. S.K.B.: acquisition of data, study design, finalized the manuscript. A.G.: formal analysis, draft writing, critical revision of manuscript. All the authors approved the submission.

## Conflicts of interest disclosure

The authors declare they have no conflict of interest.

## Research registration unique identifying number (UIN)

Our study is a systematic review and the protocol of our study is registered in PROSPERO and can be accessed with the PROSPERO : CRD42022333576.

## Guarantor

Dr Anil Bist.

## Data availability

All data generated during this study are included in this article.

Data used in the review are available in supplementary Files, Supplemental Digital Content 3, http://links.lww.com/MS9/A386.

## Supplementary Material

## References

[1] Benner A Patel AK Singh K. Physiology, Bohr Effect. [Updated 2023 Aug 8]. In: StatPearls [Internet]. Treasure Island (FL). StatPearls Publishing; 2024. https://www.ncbi.nlm.nih.gov/books/NBK526028/30252284

[2] Haynes JM. Quality assurance of the pulmonary function technologist. Respir Care 2012;57:114–122; discussion 122–6. 22222130 10.4187/respcare.01401

[3] Miller MR Hankinson J Brusasco V. American Thoracic Society, European Respiratory Society. Standardisation of Spirometry. 2005;26:319–338. 10.1183/09031936.05.00034805 16055882

[4] Lawn ND Fletcher DD Henderson RD. Anticipating mechanical ventilation in Guillain-Barré syndrome. Arch Neurol 2001;58:893–898. 11405803 10.1001/archneur.58.6.893

[5] Leuppi JD Miedinger D Chhajed PN. Quality of spirometry in primary care for case finding of airway obstruction in smokers. Respiration 2010;79:469–474. 19786731 10.1159/000243162

[6] Schermer TR Jacobs JE Chavannes NH. Validity of spirometric testing in a general practice population of patients with chronic obstructive pulmonary disease (COPD). Thorax 2003;58:861–866. 14514938 10.1136/thorax.58.10.861 PMC1746497

[7] Hnatiuk O Moores L Loughney T. Evaluation of internists’ spirometric interpretations. J Gen Intern Med 1996;11:204–208. 8744877 10.1007/BF02642476

[8] Enright PL. Should we keep pushing for a spirometer in every doctor’s office? Respir Care 2012;57:146–151; discussion 151–3. 22222133 10.4187/respcare.01504

[9] Page MJ McKenzie JE Bossuyt PM. The PRISMA 2020 statement: an updated guideline for reporting systematic reviews. Rev Esp Cardiol 2021;74:790–799. 34446261 10.1016/j.rec.2021.07.010

[10] Wells GA Shea B O’Connell D. The Newcastle-Ottawa Scale (NOS) for assessing the quality of nonrandomised studies in meta-analyses. 2013.

[11] Elsheikh B Arnold WD Gharibshahi S. Correlation of single-breath count test and neck flexor muscle strength with spirometry in myasthenia gravis. Muscle Nerve 2016;53:134–136. 26437790 10.1002/mus.24929 PMC4715713

[12] Kumari A Malik S Narkeesh K. Single breath count: a simple pulmonary function test using a mobile app. Indian J Thorac Cardiovasc Surg 2017;33:369–370.

[13] Kalita J Kumar M Misra UK. Serial single breath count is a reliable tool for monitoring respiratory functions in Guillain-Barré Syndrome. J Clin Neurosci 2020;72:50–56. 31982274 10.1016/j.jocn.2020.01.032

[14] Bartfield JM Ushkow BS Rosen JM. Single breath counting in the assessment of pulmonary function. Ann Emerg Med 1994;24:256–259. 8037392 10.1016/s0196-0644(94)70138-5

[15] Ali SS O’Connell C Kass L. Single-breath counting: a pilot study of a novel technique for measuring pulmonary function in children. Am J Emerg Med 2011;29:33–36. 20825771 10.1016/j.ajem.2009.07.006

[16] Longhitano Y Zanza C Romenskaya T. Single-breath counting test predicts non-invasive respiratory support requirements in patients with COVID-19 pneumonia. J Clin Med Res 2021;11:5: 10.3390/jcm11010179 PMC8745879 35011920

[17] Kannan Kanikannan MA Durga P Venigalla NK. Simple bedside predictors of mechanical ventilation in patients with Guillain-Barre syndrome. J Crit Care 2014;29:219–223. 24378177 10.1016/j.jcrc.2013.10.026

[18] Escóssio AL Araújo RCde Oliver N. Accuracy of single-breath counting test to determine slow vital capacity in hospitalized patients. Rev CEFAC 2019;21:e2119.

[19] Ushkow BS Bartfield JM Reicho PR. Single-breath counting for the assessment of bronchospastic patients in the ED. Am J Emerg Med 1998;16:100–101. 9451330 10.1016/s0735-6757(98)90081-x

[20] Bhandare SA Rasal SS Iyer SK. Correlation of peak expiratory flow rate and single breath count in normal adults. Med Sci Monit 2021;9:1960.

[21] Kukulka K Gummi RR Govindarajan R. A telephonic single breath count test for screening of exacerbations of myasthenia gravis: a pilot study. Muscle Nerve 2020;62:258–261. 32447763 10.1002/mus.26987

[22] Rega PP Bork CE Burkholder-Allen K. Single-breath-count test: an important adjunct in the triaging of patients in a mass-casualty incident due to botulism. Prehosp Disaster Med 2010;25:219–222. 20586014 10.1017/s1049023x00008062

[23] Quinn C Mcmillan CT Owegi MA. Single breath counting is an effective screening tool for forced vital capacity in ALS. Amyotroph Lateral Scler Frontotemporal Degener 2021;22(supp1):5–8. 34348533 10.1080/21678421.2021.1915337

[24] Menon J Joseph JK Jose MP. Management of snakebite - an update. Progress in Medicine 2017.

[25] Octaviana F Safri AY Wiratman W. Pulmonary function assessment in myasthenia gravis patients in a national referral hospital in Indonesia. Int J Gen Med 2023;16:4477–4483. 37808209 10.2147/IJGM.S426321 PMC10559783

[26] Aguirre F Fernández RN Arrejoría RM. Peak expiratory flow and the single-breath count test as markers of respiratory function in patients with myasthenia gravis. Neurologia 2023;38:405–411. 35842128 10.1016/j.nrleng.2020.09.006

[27] Dishnica N Vuong A Xiong L. Single count breath test for the evaluation of respiratory function in myasthenia gravis: a systematic review. J Clin Neurosci 2023;112:58–63. 37094510 10.1016/j.jocn.2023.04.011

[28] Shea BJ Reeves BC Wells G. AMSTAR 2: a critical appraisal tool for systematic reviews that include randomised or non-randomised studies of healthcare interventions, or both. BMJ 2017;358:j4008. 28935701 10.1136/bmj.j4008 PMC5833365

[29] Kumar S Rani P Sharma J. Guillain–Barré syndrome: clinical profile and electrodiagnostic subtype spectrum from a tertiary care hospital in eastern India. Int J Nutr Pharmacol Neurol Dis 2022;12:163–169.

