# Supplementary material for: Single breath count test and its applications in clinical practice: a systematic review
Source: Ann Med Surg (Lond). 2024 Feb 28;86(4):2130–6. doi: 10.1097/MS9.0000000000001853 (PMC10990392; doi:10.1097/MS9.0000000000001853)
Supplement: Supplementary file 3 [file ms9-86-2130-s003.docx]

**Supplementary Files:**

1. Search Strategy in EMBASE, PubMed and Google Scholar.
2. Quality Assessment of included studies by Newcastle Ottawa Scale.

**S1-Search Strategy for searching relevant articles in electronic databases.**

**PubMed: (n)=35**

((((single breath count[Title/Abstract]) OR (single-breath count[Title/Abstract])) OR (single breath count test[Title/Abstract])) OR (single breath counting[Title/Abstract])) OR (sbct[Title/Abstract]) AND ((humans[Filter]) AND (english[Filter]))

**EMBASE: (n)=85**

#7. (#1 OR #2 OR #3 OR #4 OR #5) AND [english]/lim

AND [humans]/lim

#6. #1 OR #2 OR #3 OR #4 OR #5

#5. sbct

#4. 'single breath counting'

#3. 'single-breath count test'

#2. 'single breath count test'

#1. 'single breath count'

**Google Scholar: (n)=34**

allintitle: "single breath count" OR "single-breath count test" OR "single breath count test" OR "single breath counting" OR sbct

**S2-Quality assessment by New Castle Ottawa Scale.**

| Author | Representativeness of the sample | Sample size: | Non-respondents | Ascertainment of the exposure | Comparability of subjects | Assessment of outcome | Statistical test | Total score |
| --- | --- | --- | --- | --- | --- | --- | --- | --- |
| Elshiekh et.al | No description of the derivation of the included subjects. | No information provided on sample size | Unsatisfactory recruitment rate, no summary data on non-respondents | Vaccine records/vaccine registry/clinic registers/hospital records only. ** | Not applicable | Independent blind/unblind assessment using objective validated laboratory methods. ** | Statistical test used to analyse the data clearly described, appropriate and measures of association presented including confidence intervals and probability level (p value). * | 5 |
| Kukulka et.al. | Selected group of users/convenience sample | No information provided on sample size | Unsatisfactory recruitment rate, no summary data on non-respondents | Vaccine records/vaccine registry/clinic registers/hospital records only. ** | Not applicable | Independent blind/unblind assessment using objective validated laboratory methods. ** | Statistical test used to analyse the data clearly described, appropriate and measures of association presented including confidence intervals and probability level (p value). * | 5 |
| Kumari et.al. | Truly representative of the average in the target population. * | No information provided | Unsatisfactory recruitment rate, no summary data on non-respondents. | Vaccine records/vaccine registry/clinic registers/hospital records only. ** | Data/results not adjusted for all relevant confounders/risk factors/information not provided. | Used non-standard or non-validated laboratory methods with gold standard.* * | Statistical test used to analyse the data clearly described, appropriate and measures of association presented including confidence intervals and probability level (p value). * | 5 |
| Kalita et.al. | Somewhat representative of the average in the target group. * (non-random sampling) | No information provided | No information provided | Vaccine records/vaccine registry/clinic registers/hospital records only. ** | Data/results not adjusted for all relevant confounders/risk factors/information not provided. | Unblinded assessment using objective validated laboratory methods. ** | Statistical test used to analyse the data clearly described, appropriate and measures of association presented including confidence intervals and probability level (p value). * | 6 |
| Kanikannan et.al. | Selected group of users/convenience sample | No information provided | Unsatisfactory recruitment rate, no summary data on non-respondents | Vaccine records/vaccine registry/clinic registers/hospital records only. ** | Not applicable | Unblinded assessment using objective validated laboratory methods. ** | Statistical test used to analyse the data clearly described, appropriate and measures of association presented including confidence intervals and probability level (p value). * | 5 |
| Quinn et.al. | Somewhat representative of the average in the target group. * (non-random sampling) | No information provided | No information provided | Vaccine records/vaccine registry/clinic registers/hospital records only. ** | Not applicable | Unblinded assessment using objective validated laboratory methods. ** | Statistical test used to analyse the data clearly described, appropriate and measures of association presented including confidence intervals and probability level (p value). * | 6 |
| Bartfield et.al. | Selected group of users/convenience sample. | No information provided | No information provided | Vaccine records/vaccine registry/clinic registers/hospital records only. ** | Not applicable | Unblinded assessment using objective validated laboratory methods. ** | Statistical test not appropriate, not described or incomplete. | 4 |
| Ushkow et.al. | Selected group of users/convenience sample. | No information provided | No information provided | Vaccine records/vaccine registry/clinic registers/hospital records only. ** | Not applicable | Unblinded assessment using objective validated laboratory methods. ** | Statistical test not appropriate, not described or incomplete. | 4 |
| Ali et.al. | Selected group of users/convenience sample. | No information provided | No information provided | Vaccine records/vaccine registry/clinic registers/hospital records only. ** | Not applicable | Unblinded assessment using objective validated laboratory methods. ** | Statistical test used to analyse the data clearly described, appropriate and measures of association presented including confidence intervals and probability level (p value). * | 5 |
| Escossio et.al. | Somewhat representative of the average in the target group. * (non-random sampling) | No information provided | No information provided | Vaccine records/vaccine registry/clinic registers/hospital records only. ** | Not applicable | Unblinded assessment using objective validated laboratory methods. ** | Statistical test used to analyse the data clearly described, appropriate and measures of association presented including confidence intervals and probability level (p value). * | 6 |
| Longhitano et.al. | Somewhat representative of the average in the target group. * (non-random sampling) | Not applicable | Not applicable | Vaccine records/vaccine registry/clinic registers/hospital records only. ** | Not applicable | Unblinded assessment using objective validated laboratory methods. ** | Statistical test used to analyse the data clearly described, appropriate and measures of association presented including confidence intervals and probability level (p value). * | 6 |
| Bhandare et.al. | Selected group of users/convenience sample. | Not applicable | Not applicable | Vaccine records/vaccine registry/clinic registers/hospital records only. ** | Not applicable | Unblinded assessment using objective validated laboratory methods. ** | Statistical test used to analyse the data clearly described, appropriate and measures of association presented including confidence intervals and probability level (p value). * | 5 |
| Rega et.al. | Not applicable | Not applicable | Not applicable | Not applicable | Not applicable | Not applicable | Not applicable | 0 |
